# Control of Dendritic Cell Function Within the Tumour Microenvironment

**DOI:** 10.3389/fimmu.2022.733800

**Published:** 2022-03-10

**Authors:** Yukti Hari Gupta, Abida Khanom, Sophie E. Acton

**Affiliations:** Stromal Immunology Laboratory, MRC Laboratory for Molecular Cell Biology, University College London, London, United Kingdom

**Keywords:** tumour microenvironment (TME), inflammatory cytokines, dendritic cells, anti-tumour immunity, draining lymph nodes, Tertiary Lymphoid Structures (TLS), immune infiltration

## Abstract

The tumour microenvironment (TME) presents a major block to anti-tumour immune responses and to effective cancer immunotherapy. The inflammatory mediators such as cytokines, chemokines, growth factors and prostaglandins generated in the TME alter the phenotype and function of dendritic cells (DCs) that are critical for a successful adaptive immune response against the growing tumour. In this mini review we discuss how tumour cells and the surrounding stroma modulate DC maturation and trafficking to impact T cell function. Fibroblastic stroma and the associated extracellular matrix around tumours can also provide physical restrictions to infiltrating DCs and other leukocytes. We discuss interactions between the inflammatory TME and infiltrating immune cell function, exploring how the inflammatory TME affects generation of T cell-driven anti-tumour immunity. We discuss the open question of the relative importance of antigen-presentation site; locally within the TME versus tumour-draining lymph nodes. Addressing these questions will potentially increase immune surveillance and enhance anti-tumour immunity.

## Introduction

Anti-tumour immunity is the ability of the body’s immune system to recognise and eliminate tumour cells. This phenomenon has the potential to cure cancer even if cells are widely disseminated through multiple metastatic sites and has been harnessed to develop different immunotherapy drugs. With increased understanding of immune surveillance process by innate immune cells and discovery of T cell immune checkpoints, such as PD-1, PD-L1, and CTLA-4; cancer immunotherapy has significantly improved patient survival and quality of life (1–5). Treatments aim to promote successful infiltration and activation of antigen presenting cells and boost T-cells cytotoxic activity to promote anti-tumour immunity. However, despite promising results, not all tumour types or patients respond equally to immunotherapy (6–8). The major reasons for failure of immunotherapy are (1) reduced antigenicity (9–11) and (2) immunosuppressive tumour microenvironment (TME) (12–15). The TME is highly heterogeneous; consisting of tumour cells, stromal cells, extracellular matrix (ECM) and immune cell types including macrophages, dendritic cells, T and B lymphocytes, Natural killer (NK) cells, mast cells, myeloid derived suppressor cells (MDSCs) and neutrophils (16–20). The anti-tumour immune response relies on the antigen presenting cells (APCs) to prime naïve T cells. Tissue resident macrophages can activate T cells locally in the tumour; whereas dendritic cells (DCs), the professional APCs, are thought to migrate into the tumour draining lymph nodes (TDLNs) to prime T cells (21). However, immune surveillance by APCs and T-cell infiltration can be impaired by dynamic changes within the tumour microenvironment such as induction of chemokines, cytokines, growth factors, inflammation, ECM modulators and immune checkpoint proteins (22–27). This review focuses on the immunosuppressive properties of the TME and how these mechanisms alter activation, maturation and trafficking of dendritic cells to enable immune escape and tumour progression.

### DC Maturation and DC Gene Signatures in Tumours

DCs are the professional APCs responsible for activation and maintenance of tumour-specific cytotoxicity by T cells (28, 29). Tumour infiltrating conventional DCs (cDC1 and cDC2) scan and phagocytose tumour antigens (30–32); and subsequently migrate to secondary lymphoid tissues to prime naïve CD8+ and CD4+ T cells (33–39). The phenotype and function of highly motile DCs is influenced by co-stimulatory molecules (CD80, CD86), chemokine receptors such as CCR7 and cell adhesion molecules (integrins, ICAM-1 and VCAM-1) (40–43). It has been well established that the interaction between CC chemokine receptor 7 (CCR7) upregulated on activated DCs and its ligand CC chemokine ligand 21 (CCL21) expressed by lymphatic endothelial cells (LECs) is essential for directional DC migration towards the lymph nodes (44–46). Upon entry to the LN, DCs use the C-type lectin CLEC-2 to migrate through the fibroblastic reticular network to reach the paracortex to stimulate the T cells (47–50). Secondary lymphoid tissues are structurally specialised to facilitate effective adaptive immune responses; however, the microenvironment of the tumour-draining lymph nodes (TDLNs) can be immune-suppressed in cancer patients and can display low DC count, defects in DC development, low levels of costimulatory molecules or accumulation of immature T cells (51, 52). DCs evaluated from TDLNs of an immunized B16F10 melanoma-bearing mice showed decreased functionality and expressed higher CD86 and lower CD206 levels (53). Similarly, in a study by Caronni et al., LNs draining lung tumours exhibited DCs with reduced antigen presentation due to downregulation of the SNARE VAMP3 and failed cytokine (IL12 and IFN-I) secretion. They reported lactic acid formation in the TME to be the main cause of DC function impairment (54). In addition, damage-associated molecular patterns (DAMPs) released from dying cells in the TME can also influence dendritic cells and other immune cells by interacting with toll-like receptors (TLRs) contributing to immunosuppressive phenotype (55). Lack of mature, migratory DCs in tumours correlates with poor prognosis in cancer patients and failure of immunotherapies (56–58). Recent development of single cell transcriptome profiling of tumour infiltrating DCs has proven to be a very powerful tool to map tumour-driven immune changes and to design future immune therapies leveraging DC biology. scRNA-seq studies on various human tumours, including non–small cell lung cancer (NSCLC) (59–62), head and neck squamous cell carcinoma (63), hepatocellular carcinoma (64), melanoma (65, 66), cutaneous squamous cell carcinoma (67), colorectal cancer (61, 68), ovarian cancer (61), and breast cancer (61) have identified tissue-specific DC subsets as well as those conserved across cancer types. By comparing tumour infiltrating DC states across various tumour studies, five major DC subsets have been defined that are conserved in most tumour types (69, 70) (**Table 1**). Four major ones are cDC1, cDC2, migratory DC3 (mDC3) and plasmacytoid DC (pDC); and the DC subset (DC5) that were less conserved, mostly contained cDC2 state (CD1C+) but additionally either expressed Langerhans cell-specific markers (CD201, CD1A) or monocyte markers (CD14, CD11b) such as in case of NSCLC (61, 62, 69, 70). DC5 were also referred as inflammatory DCs as these have phenotypic similarities to monocytes but are functionally different due to their cDC2-specific antigen presentation properties (71). On the other hand, classical monocytes (CD14+ CD16-) play a key role in tissue homeostasis and inflammation (72). Like monocytes, inflammatory DCs are also capable of releasing TNF-α and inducible nitric oxide synthase (iNOS) upon pathogen recognition. In addition, there is a subset of cDCs that induce antigen-specific tolerance in dLNs; known as regulatory DCs (DCregs) (73, 74). These are characterized by low MHC expression and therefore weaker antigen presentation capability to effector T cells. Instead, they can induce proliferation of regulatory T cells (Tregs) resulting in immune tolerance. These properties have led the use of DCregs in organ transplantations (75).

**Table 1:** Tumour infiltrating DC subsets detected in various human solid tumours – Liver, Ovarian, Lung, Breast and Colorectal (69, 70).

DC subsets	Markers
cDC1	XCR1, CLEC9A, CADM1, CD141, CD103
cDC2	CD11b, SIRPa, CLEC10A, FCER1A, CD1c
mDC3	MARCKS, CCL19, LAMP3, BATF3, CCR7, CD40
pDC	TCf4, CXCR3, LILRA4, CLCEC4C, IRF7
DC5 or inflammatory DCs	CD1c, CD201, CD1A, CD14

Overall cDC2 phenotype is the most abundant, while the other DC subtypes vary in each cancer type (61, 76). Single cell sequencing and clustering analysis have identified transcription factors underlying each DC phenotype, including *BATF3* for cDC1s, *CEBPB* for cDC2s, *NFKB2* for migratory cDCs and *TCF4* for pDCs (61, 77). Another study reports differential expression of costimulatory molecules and immune checkpoints on different DC subsets present in the TME (78). Although these phenomena are tightly regulated, heterogeneity of TME can influence the transcriptional factor activity, expression of costimulatory molecules and hence DC maturation and/or migration (78–82). This new in-depth knowledge of DC gene signatures can facilitate the design of a favourable antitumour response or identification of response biomarkers for targeted therapies (83).

## TME Factors Affecting DC Development in Tumours

### Pro- and Anti-Inflammatory Factors

**Figure 1:**
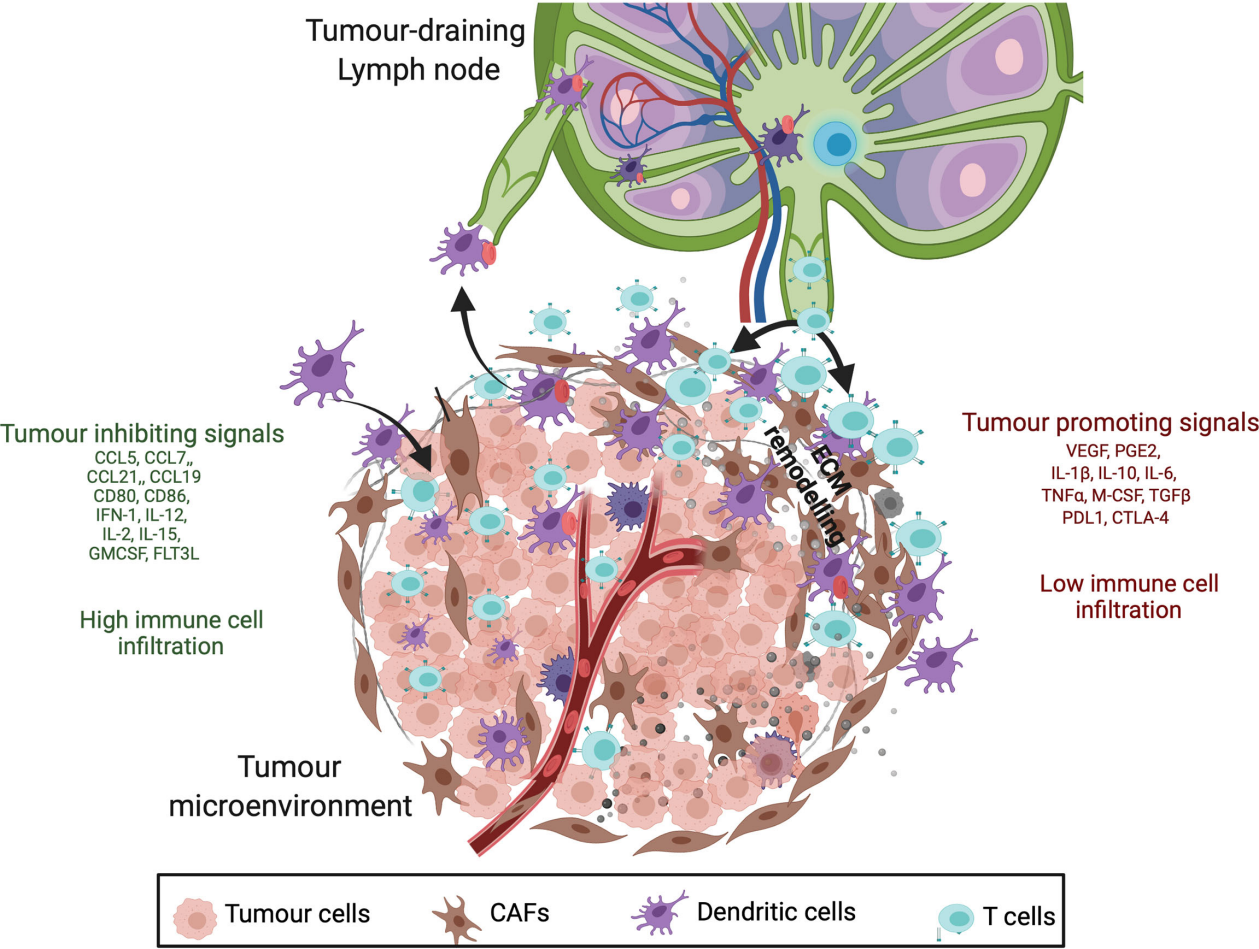
Cancer inhibitory and cancer-promoting signals within the tumour microenvironment (TME). Anti-tumour response is initiated by antigen recognition and trafficking by mature DCs to the tumour draining lymph node (TDLN) which involves upregulation of chemokine receptors (CCR7), MHC class II, co-stimulatory molecules (CD80 and CD86), inflammatory molecules (IL-12, INF-1) and adhesion molecules (ICAM-1) (listed in green). Having said that, immunosuppressive nature of TME secretes tumour promoting inflammatory mediators (listed in red) such as prostaglandin E2, cytokines (IL-10, IL-6, TGFß), chemokines (CXCL1) and growth factors (VEGF) that impede anti-tumour response by altering DC phenotype, T-cell infiltration and ECM remodelling. These differences result in poor surveillance by DCs and lower infiltration of T cells in tumours with immunosuppressive molecules (red).

The immunosuppression of tumour-infiltrating DCs can be facilitated by various soluble factors secreted in the TME such as IL-6, IL-10, IDO, M-CSF, transforming growth factor-β1 (TGF-β1), PGE2, VEGF (**Figure 1**) (84–91); although promisingly some of these defects in DC development or function have been proven to be reversible in pre-clinical models and clinical trials (27, 91–94). Mature DC numbers or functions were improved leading to better immune control of the tumour in several mouse models: IL-6 KO mice (95); tumours treated with anti-VEGF antibody (96, 97); and treatment with anti-IL-8 monoclonal antibody (98, 99). On the other hand, pro-inflammatory cytokines such as IFN-α, IL-2, IL-15, IL-21 and GM-CSF are also present in the TME (**Figure 1**) that contribute to enhanced antigen priming, improved DC maturation and increased immune infiltration in tumours (100–103). Therefore, the complex balance of inflammatory signals in the TME is an area of intense research interest but is not trivial to target currently. One of the recent studies on human melanoma reported the correlation of pro-inflammatory cytokine FLT3L production (by NK cells) with abundant intratumoral stimulatory DCs, improved patient responsiveness to anti-PD-1 therapies and better overall survival (104).

The inflammatory factors described above can be derived from tumour cells, immune cells or stromal cells such as fibroblasts surrounding tumour (61, 88, 105, 106). Various subtypes of fibroblasts based on different tissue specific identity, localization, function, transcription factor expression, collagen factors, cancer hallmark genes etc. make up the total tumour mass. CAFs or cancer associated fibroblasts represent a major population in the TME of many solid tumours, however their origin and role in tumour progression is complex and they can generate pro-tumourigenic and anti-tumourigenic secretory factors. Phenotypically and functionally different CAF subtypes based on cell-surface markers such as podoplanin (PDPN), α-smooth muscle actin (αSMA), fibroblast-activated protein (FAP), fibroblast-specific protein-1 (FSP-1/S100A4), THY1 (also known as CD90), and platelet-derived growth factor receptor-α, and β (PDGFRα and PDGFRβ) have been associated with different tumour types, stages and patient survival (107–111). Recently, the ability of CAFs to modulate the immune responses has been discovered and is being explored to improve cancer therapies. CAFs also share some properties with fibroblasts in lymph nodes that already have a well-established role in DC migration (47, 112, 113); and therefore, parallels can be drawn between the two to better understand the DC trafficking in the TME. For example, PDPN present in fibroblasts interacts with CCL21 and promotes CCL21/CCR7 axis mediated DC migration in lymph node. This knowledge was exploited to study the role of PDPN+ CAFs under the influence of hypoxia in tumour progression (114). The study reported PDPN overexpression due to hypoxia in fact favoured invasion of CCR7+ tumour cell into CCL21+ peripheral lymph nodes leading to metastasis (114, 115). Tumours associated with hypoxia are immunosuppressive and lack high expression of CCL21 and therefore therapeutic use of recombinant chemokines (such as CCL21) to stimulate immune cell recognition in tumours is being considered as a novel treatment approach (116, 117). Also, more research is required to understand the transition of a ‘normal’ fibroblast into an immunosuppressive phenotype such as S100A4^+^ PDPN^+^ CAFs as reported in breast cancer patients (109) or into an inflammatory CAF (iCAF) phenotype producing IL-6, IL-10, and IDO (118, 119) linked to poor patient survival. Authors of Fang et al. (118) have shown the role of the urokinase-type plasminogen activator, PLAU in conversion of fibroblasts to iCAFs in esophgeal cancer (118), but much is still unknown about fibroblast differentiation in TME.

## Tertiary Lymphoid Structures (TLS)

**Figure 2:**
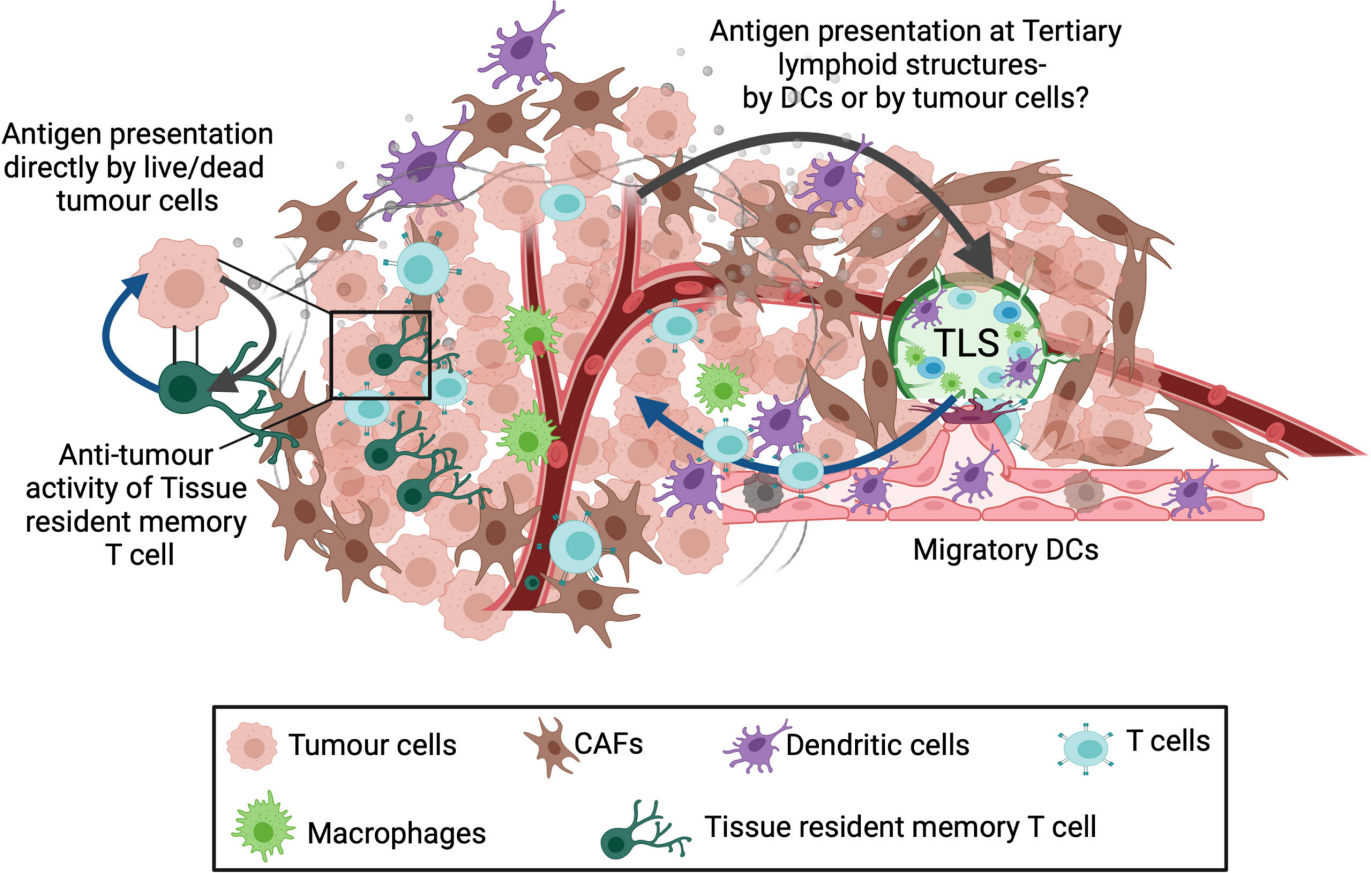
Alternate sites of antigen presentation and T-cell priming. Three different sites for presentation of tumour associated antigens have been described: Tumour draining lymph node (TDLN), Tertiary lymphoid structures (TLS) and Tissue resident memory T cells. A population of memory precursor cells are believed to differentiate into CD103+ tissue resident memory T cells. These cells reside in the tumour and can recognize tumour antigens followed by killing the target tumour cell. In addition, tertiary lymphoid structures (TLS) also present a potential site for T cell priming. TLSs are organised cell aggregates formed within or at tumour margins in response to local inflammation and numerous cell-cell interactions occurring within the TME. Since these contain various immune cell types, TLSs can activate local immune response against the tumour, however the mechanism for T-cell priming within the TLSs is unknown.

TLS are established at sites of chronic inflammation and can structurally and functionally resemble secondary lymphoid organs (120–122). Recent studies on murine models of TLS have shown the role of PDPN+ FAP+ immunofibroblasts in driving the development and expansion of TLSs (123, 124). These form part of the TME and can benefit from quick surveillance and locally primed immune response against tumour antigens (**Figure 2**). Occurrence of TLS correlated with high number of mature DCs, strong T-cell infiltration and long-term survival in human primary lung, breast, colorectal, melanoma and other tumours (120, 125–128). However, factors such as TLS location, tumour stage, tumour mutations, treatment history can affect immune cell infiltration and anti-tumour response (128, 129). The cells residing in TLS in tumours are known to express Th1, CD4, CD8, CD31, CD23, FOXP3, chemokines (CCL19, CCL21) and clusters of DC-Lamp^+^ mature dendritic cells (120, 130, 131) providing an immune-supportive niche (132–134). Typically, TLS at the periphery of the tumour have more organised and distinct DC/T-cell and B-cell zones than intratumoral TLS which contain mostly B cells (133). Future research understanding the immunological features of extratumoral versus intratumoral TLS will be useful to predict responsiveness to immunotherapy and overall survival.

## Immune Checkpoint Genes

The other group of molecules responsible for causing dysfunction in tumour-infiltrating DCs are immune checkpoint proteins PD-L1, PD-1, ILT2, CTLA4, TIM3 expressed by tumour cells or other immune cells (135–141). As mentioned before, expression of these inhibitory molecules is variable among DC subsets. For example, PD-1 and TIM-3 are mostly expressed on cDC1s; PD-1 expression specifically has been shown to inhibit NF-kB activation which is critical for DC functions including costimulatory molecule expression, antigen presentation and cytokine release leading to T cell inactivation (78, 135, 137, 139, 140). On contrary, ILT2 is expressed on pDCs and cDC2s, but not on cDC1s (78). The central goal of immunotherapies is inhibition of immune checkpoint genes and the expansion of mature cDCs and cytotoxic CD8+ T cells within tumours. It is associated with positive patient outcomes in multiple cancer types when combined with chemotherapy or radiotherapy treatments (28, 135, 142, 143). Despite this, many patients still fail to respond to immune checkpoint blockade. A better understanding of the role of inflammatory mediators in determining tumour progression will also provide therapeutic avenues to improve immunotherapy outcomes (144–147).

Different labs have reported direct inhibition of pro-tumourigenic inflammation in combination with immune checkpoint blockade as a powerful strategy to improve the patient survival rates (27, 148–150). One such example is the use of aspirin that blocks the COX-2/PGE2 pathway and has shown promising results in preclinical melanoma models (27, 149). Prostaglandin E2 (PGE2), catalysed by the enzyme COX-2 is elevated in many tumours (151) and plays a role in tumour evasion by directly inhibiting cytotoxic immune responses and subsequently mediates expression of other inflammatory molecules such as CXCL9, CXCL10, CXCR4, CXCL12, IDO1 and interferon (IFN)-γ (27, 144, 148, 150, 152–154). Induction of CXCL12, CXCR4 and IDO1 in tumours have been associated with accumulation of myeloid derived suppressor cells (90, 155). Moreover, direct interaction of EP2/EP4 receptors (present on DCs) with the available PGE2 can modulate DC maturation, metalloprotease-driven DC motility, and immune response in tumours (27, 149, 152, 156–158). Thus, targeting the inflammatory environment of the tumour is important to restore DC function to harvest the full potential of immunotherapy.

## Leveraging DC Biology in Cancer Therapies

Anti-tumour immunity relies on cross-presentation of tumour antigens by DCs to elicit a CD8+ T cell response. Among various DC subsets, cDC1s (XCR1+, CD103+) play a critical role in anti-tumour immunity. CLEC9A, (also known as DNGR1) is highly expressed on cDC1s and binds necrotic cell debris and promotes antigen processing in tumours (159–161). One of the reasons for checkpoint blockade failure is poor antigen presentation due to absence of co-stimulatory molecules and therefore modulation of DC function could increase responses to these therapies. One method to address this issue is the development of DC vaccines for cancer treatment, bypassing the need to activate and mature DCs within the tumour. DC-based cancer vaccines work by recruiting ex-vivo generated dendritic cells (or monocyte derived patient DCs) that are genetically engineered, matured, and loaded with tumour-specific antigens (162–164) or by reprogramming endogenous DCs by injecting biomaterial-based scaffolds providing favourable microenvironment for the recruitment of activated DCs (165, 166). An ideal DC vaccine must be able to increase cross-presentation by DCs, express high levels of co-stimulatory molecules, induce tumour-specific T cells with high migratory and cytolytic capabilities. Furthermore, the use of dendritic growth factor Flt3L in combination with checkpoint inhibitors or DC vaccines has improved number of activated intratumoural cDC1s and enhanced anti-tumour immunity to BRAF and checkpoint blockade in preclinical models (167–170).

Presence of co-inhibitory signals (e.g., IL-10, IL-6, PGE2, TGF-β) or absence of co-stimulatory molecules (e.g. CD80 and CD86) can result in inefficient antigen presentation by DCs and poor induction of antigen-specific CD8+T cells. Therefore, inflammatory cytokines secreted by tumour cells and tumour-associated stroma have been identified as promising candidates to potentiate current immunotherapies including immune checkpoint blockade and CAR-T therapy (149, 171–173). Stroma present around most tumours can also magnify inflammation and impede DC phenotype (174–177) and hence manipulating stroma/DC crosstalk in the TME could help improve DC function.

## Discussion

It is now established that tumours can exploit their surroundings to create an immunosuppressive microenvironment to control DC function within both the TME and TDLNs (178, 179). These signals including cytokines, chemokines, prostaglandins, growth factors, immune checkpoint genes, etc., may target different DC subsets infiltrating tumours and influence DC maturation, antigen uptake and DC migration (53, 180). Although the success of immunotherapy relies on enhanced T cell activity, activation of tumour-specific T cells cannot be achieved without prior antigen presentation by professional DCs. To overcome immunosuppressive signals, personalized vaccines loaded with patient-derived engineered DCs or delivery of innate stimulus such as TLR3 ligand or a STING agonist to DCs at the tumour site are being developed and have shown promising results (181, 182). Repurposing of existing anti-inflammatory drugs such as aspirin along with DC vaccines or immunotherapies has also been successfully tested in pre-clinical models (149).

This review also addresses the importance of local versus TDLN priming of anti-tumoural T cell responses. Tissue resident memory CD103+ CD8^+^ T cells residing in the non-lymphoid tissues have shown to provide local immunosurveillance and enhanced immune responses in melanoma, lung and breast tumours (183–187). Moreover, melanoma patients with higher resident T cell population responded better to anti-PD-1 immunotherapy with improved survival (188, 189). However, what is still unclear is how are tissue resident memory CD8^+^ T cells primed (**Figure 2**) and whether there is a distinct population of DCs required to activate them. Although the exact regulatory mechanisms remain to be explored further, it is hypothesized that crosstalk between tissue resident memory T cells, tumour cells, stromal cells and DCs within the TME potentiate secondary T-cell responses against tumours (**Figure 2**). This also opens discussion on the role of tumour associated tertiary lymphoid structures (TLSs) in intra-tumoural DC maturation; and sourcing T cells and B cells to the tumour (190). Although TLS has been positively correlated with anti-tumour responses, there are still many questions remain to be answered such as TLS composition and TLS induction at tumour site before TLS can be adopted as a predictive tool or as a therapeutic option. Our discussion demonstrates the importance of site of antigen presentation in DC maturation and trafficking which must be exploited therapeutically to enhance immune response against cancer.

## Author Contributions

YG and SA planned the concept and design of the review. YG and AK collected previous literature on the topic and drafted the article. YG made the figures. SA performed critical revision of the article. YG and SA edited the final version of the article.

## Funding

This work is funded by Cancer Research UK (Career development fellowship CRUK-A19763 to SA) and Medical Research Council (MC-U12266B).

## Conflict of Interest

The authors declare that the research was conducted in the absence of any commercial or financial relationships that could be construed as a potential conflict of interest.

## Publisher’s Note

All claims expressed in this article are solely those of the authors and do not necessarily represent those of their affiliated organizations, or those of the publisher, the editors and the reviewers. Any product that may be evaluated in this article, or claim that may be made by its manufacturer, is not guaranteed or endorsed by the publisher.

## Abbreviations

APC, Antigen Presenting cells; LNs, Lymph nodes; TDLN, Tumour draining lymph node; TME, Tumour microenvironment; DCs, Dendritic cells; PGE2, Prostaglandin E2; ECM, Extracellular matrix; CAF, Cancer associated fibroblasts; TLS, Tertiary lymphoid structures.
